# MicroRNA-146a Serves as a Biomarker for Adverse Prognosis of ST-Segment Elevation Myocardial Infarction

**DOI:** 10.1155/2021/2923441

**Published:** 2021-10-25

**Authors:** Shengjue Xiao, Tongneng Xue, Qinyuan Pan, Yue Hu, Qi Wu, Qiaozhi Liu, Xiaotong Wang, Ailin Liu, Jie Liu, Hong Zhu, Yufei Zhou, Defeng Pan

**Affiliations:** ^1^Department of Cardiology, The Affiliated Hospital of Xuzhou Medical University, Xuzhou, Jiangsu, China 221004; ^2^Department of Cardiology, Department of Cardiology, The Affiliated Huai'an No.1 People's Hospital of Nanjing Medical University, Huai'an, China 223300; ^3^Department of General Practice, The Affiliated Hospital of Xuzhou Medical University, Xuzhou, Jiangsu, China 221004; ^4^Shanghai Institute of Cardiovascular Diseases, Zhongshan Hospital and Institutes of Biomedical Sciences, Fudan University, Shanghai 200032, China

## Abstract

**Objective:**

This study is aimed at exploring the underlying molecular mechanisms of ST-segment elevation myocardial infarction (STEMI) and provides potential clinical prognostic biomarkers for STEMI.

**Methods:**

The GSE60993 dataset was downloaded from the GEO database, and the differentially expressed genes (DEGs) between STEMI and control groups were screened. Enrichment analysis of the DEGs was subsequently performed using the DAVID database. A protein–protein interaction network was constructed, and hub genes were identified. The hub genes in patients were then validated by quantitative reverse transcription-PCR. Furthermore, hub gene-miRNA interactions were evaluated using the miRTarBase database. Finally, patient data on classical cardiovascular risk factors were collected, and plasma microRNA-146a (miR-146a) levels were detected. An individualized nomogram was constructed based on multivariate Cox regression analysis.

**Results:**

A total of 239 DEGs were identified between the STEMI and control groups. Expression of S100A12 and miR-146a was significantly upregulated in STEMI samples compared with controls. STEMI patients with high levels of miR-146a had a higher risk of major adverse cardiovascular events (MACEs) than those with low levels of miR-146a (log-rank *P* = 0.034). Multivariate Cox regression analysis identified five statistically significant variables, including age, hypertension, diabetes mellitus, white blood cells, and miR-146a. A nomogram was constructed to estimate the likelihood of a MACE at one, two, and three years after STEMI.

**Conclusion:**

The incidence of MACEs in STEMI patients expressing high levels of miR-146a was significantly greater than in those expressing low levels. MicroRNA-146a can serve as a biomarker for adverse prognosis of STEMI and might function in its pathogenesis by targeting S100A12, which may exert its role via an inflammatory response. In addition, our study presents a valid and practical model to assess the probability of MACEs within three years of STEMI.

## 1. Introduction

ST-segment elevation myocardial infarction (STEMI) is a serious form of coronary heart disease associated with high rates of death and disability (1). The current protocol for detecting a myocardial infarction uses hsTnI, which is a high-sensitivity troponin I (2, 3). However, there is a lack of prognostic biomarkers for STEMI. Therefore, in order to provide a more accurate prognosis for STEMI patients, new blood-borne biomarkers need to be identified.

MicroRNAs (miRNAs) are a class of small noncoding RNAs that exist not only in prokaryotic cells, but also in large numbers in many eukaryotic organisms, and regulate 30% of genes following transcription in eukaryotic organisms (4). Increasing numbers of studies suggest that some miRNAs increase the likelihood of STEMI by regulating signaling pathways (5, 6). Currently, a number of studies have confirmed that miRNAs are involved in coronary atherosclerosis, acute myocardial infarction, myocardial fibrosis after infarction, and cardiac remodeling, among other cardiovascular conditions (7–9). In addition, miRNAs play an important role in normal and pathophysiological processes in the heart, such as cardiac development, arrhythmia, heart failure, cardiac hypertrophy, and myocardial injury (10–12). Changes in the miRNA expression are closely related to disease or injury. Hence, miRNA levels in the blood can be used as biomarkers to evaluate and monitor pathophysiological states (13).

Over the past decade, with the rapid development of bioinformatics, including microarray and sequencing for gene detection and proteomics for protein detection (14, 15), much progress has been made in the exploration of novel biomarkers for cancer and neurological, respiratory, and cardiovascular diseases (16). Therefore, in this study, we analyzed microarray data from the peripheral blood of healthy controls and STEMI patients who visited the emergency department within 4 hours after onset of chest pain. We further explored the interactions between miRNAs and genes using the miRtarbase database. Expression of S100 calcium binding protein A12 (S100A12) and miR-146a was substantially higher in STEMI samples than in control samples. In addition, we constructed a nomogram based on Cox regression to formulate prognoses for patients with STEMI, leading to personalized treatment and assisting physicians in predicting poor patient outcomes.

## 2. Materials and Methods

### 2.1. Microarray Data

**Figure 1:**
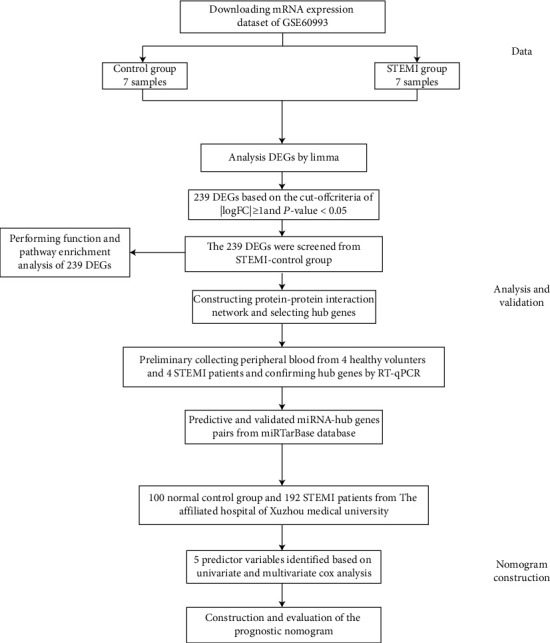
Study flow chart. Abbreviations: STEMI: ST-segment elevation myocardial infarction; DEGs: differentially expressed genes.

A workflow for this study is presented in Figure 1. GSE60993 datasets were obtained from the Gene Expression Omnibus (GEO) database (http://www.ncbi.nlm.nih.gov/geo), which is based on the GPL6884 Illumina HumanWG-6 v3.0 expression BeadChip (17). The dataset included peripheral blood from healthy controls (HCs) (*n* = 7) and patients with STEMI (*n* = 7) who visited the emergency department within 4 hours of the onset of chest pain.

### 2.2. Identification of DEGs

The GSE60993 gene expression profiles were processed with ActivePerl software to convert the gene probe IDs to gene symbol codes. Probe sets without corresponding gene symbols were removed, and genes with more than one probe set were averaged. Furthermore, the DEGs between patients with STEMI and HCs were screened using the “limma” package of *R* software. A gene expression value of the ∣log_2_ (fold change) | >1 and *P* value <0.05 was set as cut-off criteria for the STEMI DEGs. Volcano maps and heatmaps of DEGs were constructed.

### 2.3. Functional and Pathway Enrichment Analysis

We assessed Gene Ontology (GO) terms in biological processes (BP), cellular components (CC), and molecular functions (MF). In this study, the DAVID online tool was used to perform GO functional enrichment analyses and Kyoto Encyclopedia of Genes and Genomes (KEGG) pathway analyses of DEGs (18). A *P* value <0.05 and gene count ≥ 2 were set as cut-off criteria for GO terms and KEGG pathways of biological functions.

### 2.4. Construction of Protein-Protein Interaction (PPI) Networks

The Search Tool for the Retrieval of Interacting Genes/Proteins (STRING) database (http://stringdb.org/) consolidates known data from many organisms and can be used to predict and track protein–protein interactions (19). DEGs were uploaded to the STRING database, and a PPI network was established with the minimum required interaction score set at medium confidence (>0.4). Subsequently, the PPI network was visualized with CytoScape (20).

### 2.5. Initial Identification and Validation of Hub Genes and miRNAs

For the purpose of validating the expression of hub genes in humans, we collected peripheral blood from 4 STEMI patients who visited the emergency department of the Affiliated Hospital of Xuzhou Medical University within 4 hours of the onset of chest pain. In addition, 4 healthy volunteers matched by age and sex were also recruited. None of the participants had a history of cancer, autoimmune disorders, abnormal renal/liver function, homeopathy, or thyroid dysfunction as determined by their medical history, questionnaires, or clinical examination. The miRTarBase is an experimentally validated database of microRNA–target interactions (21). Predictive and validated miRNA-hub gene pairs were extracted from the miRTarBase database (21). Quantitative reverse transcription-PCR (RT-qPCR) was used to validate hub genes in the PPI network and two microRNAs (miR-146a and miR-146b). For more information on individual clinical data, see Supplementary Table S1. The research proposal was approved by the Ethics Committee of the Affiliated Hospital Xuzhou Medical University (No: XYFYLW2017-002).

### 2.6. RT-qPCR

Total RNA from blood samples was obtained using TRIzol Reagent (Invitrogen, USA). Subsequently, a cDNA synthesis kit (TIANGEN, China) was used to reverse isolated RNA into first-strand cDNA according to the manufacturer's instructions. Each 20 *μ*l PCR reaction solution contained 2 *μ*l synthesized cDNA used as the template for RT-qPCR. Specific primers were designed by PREMIER Biosoft, Inc. and are shown in Supplementary Table S2. RT-PCR was carried out on an ABI Prism 7500 sequence-detection system (Applied Biosystems, USA), and experimental data were calculated as 2^–*ΔΔ*CT^. The amplification procedure consisted of initial denaturation at 95°C for 15 min, followed by 40 PCR cycles. The predetermined cycle parameters were denaturation at 95°C for 10 s followed by annealing and extension at 60°C for 32 s.

### 2.7. Study Population

A total of 356 participants (100 HCs and 256 STEMI patients) were recruited from the Affiliated Hospital of Xuzhou Medical University from August 2017 to December 2020. Acute STEMI was defined according to the Fourth Universal Definition of Myocardial Infarction (2018) (22). There were 4 inclusion criteria for the STEMI group: (1) patients with obvious clinical symptoms of myocardial ischemia, such as chest pain, chest tightness, or shortness of breath, whose attack duration exceeded 30 minutes, and could not be completely relieved by nitroglycerin; (2) an ECG showed at least two consecutive anterior leads or at least two adjacent limb leads, ST segment elevation of 0.1 mV, or (possibly) new left bundle branch block, with a dynamic evolution of myocardial ischemia; (3) elevation of myocardial enzymes exceeded the upper 99th percentile of the reference value and exhibited dynamic evolution; and (4) coronary angiography confirmed target vessel stenosis. Inclusion criteria for the HC group included either coronary artery stenosis of less than 30% as shown by computed tomography angiography (CTA) or coronary angiography (CAG) in the same period. All subjects with neoplasm, autoimmune disorder, abnormal renal or liver function, thyroid dysfunction, congenital heart disease, chronic obstructive pulmonary disease, respiratory failure, tumors, recent infection, rheumatic heart disease, peripheral vascular disease, blood and immune system disease, or recent history of trauma were excluded. All participants completed a questionnaire to assess their eligibility for clinical research. Supplementary File S3 provides the inclusion and exclusion criteria for the STEMI patients and HCs. The questionnaire is provided in Supplementary File S4.

### 2.8. Data Collection, Clinical Endpoint, and Definitions

After careful review of medical charts, patient demographic characteristics including age, sex, smoking history, body mass index (BMI), and previous diseases (such as hypertension and diabetes mellitus (DM)) were extracted from electronic medical records. Laboratory assessments consisted of measurements of white blood cells (WBCs), hemoglobin, high-sensitivity C-reactive protein (hs-CRP), total cholesterol (TC), triglyceride, lipoprotein (a) (LP(a)), low-density lipoprotein-cholesterol (LDL-C), high-density lipoprotein-cholesterol (HDL-C), lactate dehydrogenase (LDH), creatine kinase (CK), creatine kinase MB (CKMB), hypersensitive troponin T (hsTnT), and N-terminal probrain natriuretic peptide (NT-proBNP). In addition, we obtained peripheral blood and detected expression of miR-146a in patients with STEMI within 4 hours (T4h) of the onset of chest pain, immediately postoperation (IP), and 24 hours (T24h), 72 hours (T72h), and 1 week (T1W) after STEMI. The end point was a MACE. The occurrence of MACEs in STEMI patients in hospital and up to 3 years after discharge was followed up by recording clinical data, outpatient reexamination results, or telephone follow-up during hospitalization. MACEs included cardiac death, angina, heart failure, malignant arrhythmias, and rehospitalization due to coronary heart disease. The incidence of MACEs in STEMI patients within 3 years of diagnosis was followed by outpatient review or telephone communication.

### 2.9. Statistical Analyses

Normally distributed data are reported as mean ± standard deviation, data not following a normal distribution is reported by the median, and categorical variables are reported as percentages. After tests of normality and homogeneity of variance, an independent-sample *t*-test was used to compare values between two groups. The median grouping method was used to categorize plasma miR-146a levels from 192 patients with STEMI at peak time into two groups: high expression and low expression, and Kaplan–Meier univariate analysis was used to compare the cumulative MACE rates of STEMI patients with miR-146a expression. The “rms” package of *R* software (http://www.r-project.org) was then used to create a nomogram based on multivariate Cox regression analysis. Overfitting bias was reduced by performing concordance index (C-index) and calibration analyses on 1,000 bootstrap samples. Statistical analyses were carried out using SPSS version 22.0 (SPSS Inc., Chicago, IL, USA), Prism 7.0 (GraphPad, San Diego, CA, USA), and *R* version 3.6.4 (R Foundation for Statistical Computing, Vienna, Austria). A *P* value <0.05 was considered significant.

## 3. Results

### 3.1. Identification of DEGs

**Figure 2:**
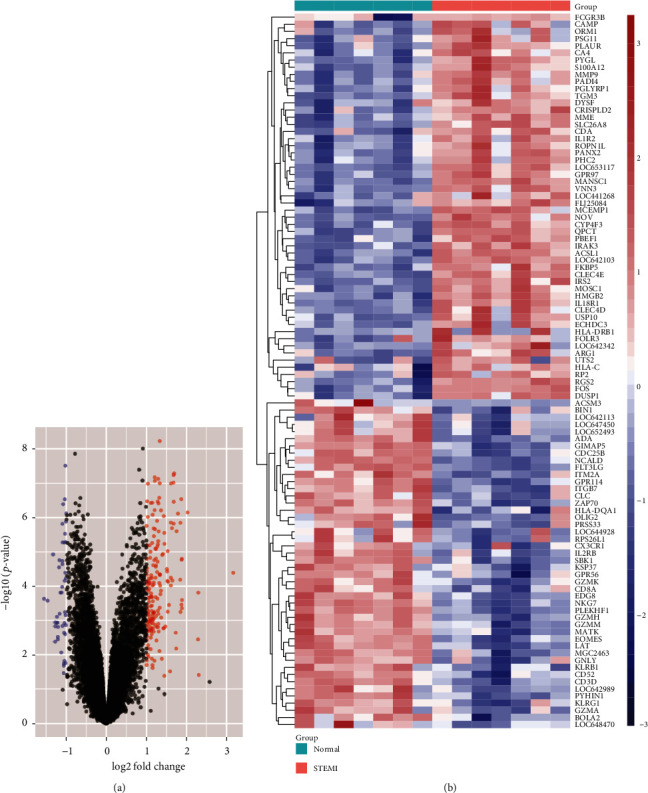
Identification of DEGs. (a) Volcano plots of the DEGs screened from the STEMI-control group. Red dots and blue dots represent upregulated and downregulated genes, respectively. (b) Cluster heat maps of the DEGs. Each row represents a sample, and each column represents a single gene. Red represents STEMI samples, and green represents healthy controls. The color scale shows relative gene expression; blue indicates low relative expression while red indicates high relative expression. Abbreviations: DEGs: differentially expressed genes; STEMI: ST-segment elevation myocardial infarction.

A total of 239 DEGs, including 193 upregulated and 46 downregulated genes, were screened between STEMI patients and healthy controls (Figure 2(a)). Cluster heatmaps of DEGs are shown in Figure 2(b), and all DEGs are displayed in Supplementary Table S5.

### 3.2. Functional Enrichment Analysis

**Figure 3:**
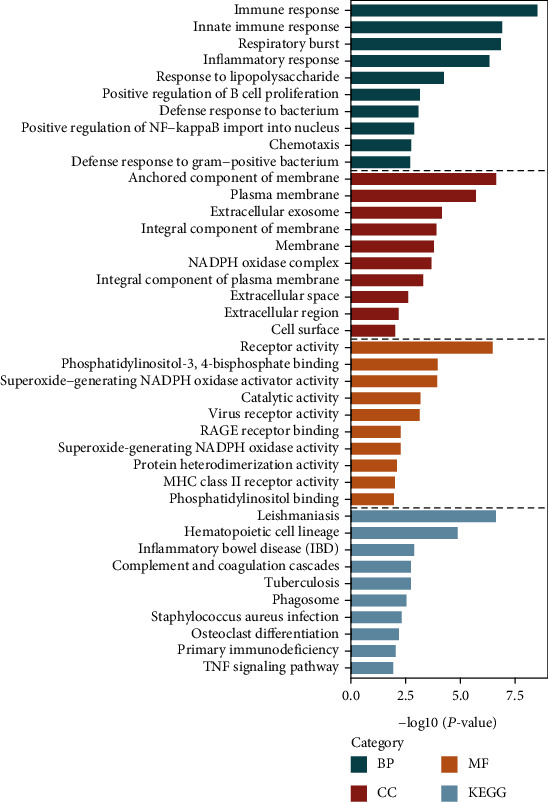
Functional enrichment analysis of DEGs. Green bars represent BPs, red bars CCs, brown lines MFs, and purple lines KEGG pathways. Abbreviations: DEGs: differentially expressed genes; BP: biological process; CC: cellular component; MF: molecular function; GO: Gene Ontology; KEGG: Kyoto Encyclopedia of Genes and Genomes.

Analyses using the DAVID database showed that DEGs were enriched mainly in BPs, including “immune response,” “innate immune response,” and “inflammatory response.” With regard to CC, DEGs were enriched in “anchored component of membrane,” “plasma membrane,” and “extracellular region.” With respect to MF, the DEGs were significantly enriched in “receptor activity,” “phosphatidylinositol-3,4-bisphosphate binding,” and “superoxide-generating NADPH oxidase activator activity” (Supplementary Table S6). Pathway enrichment-analyses using the DAVID database showed that DEGs were expressed mainly in the “Complement and coagulation cascades” and “T cell receptor signaling pathway” (Figure 3 and Supplementary Table S7).

### 3.3. Analyses of PPI Networks

**Figure 4:**
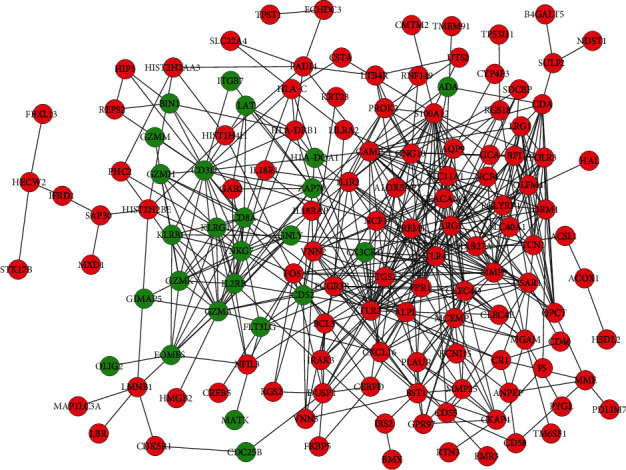
PPI network for the DEGs. Red nodes represent the upregulated DEGs, and green nodes represent downregulated DEGs. Abbreviations: PPI: protein-protein interaction; DEGs: differentially expressed genes.

For DEGs with a combined interaction score > 0.4, the STRING database was utilized to construct a PPI network with 239 intersecting DEGs. After removal of isolated nodes, the PPI network was constructed with 139 nodes and 484 edges using CytoScape (http://www.cytoscape.org/; Institute for Systems Biology, Seattle, WA) (Figure 4). Using three algorithms (Betweenness Centrality, Closeness Centrality, and Degree), the cytoHubba plug-in was applied to select hub genes of the PPI network. The top 15 genes in common identified by the three algorithms were deemed as hub genes, and a Venn diagram was constructed.

### 3.4. Validation of Hub Genes and miRNA

**Figure 5:**
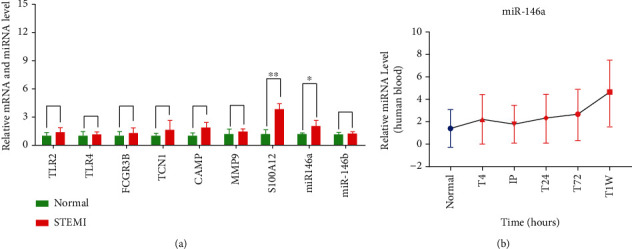
The results of verification experiments. (a) Relative expression of TLR2, TLR4, FCGR3B, CAMP, MMP9, GZMA, S100A12, miR-146a, and miR-146b were verified by RT-qPCR. ^∗^*P* < 0.05, ^∗∗^*P* < 0.01. (b) Validation of miR-146a at different time points (T4, IP, T24, T72, and T1W) in STEMI patients versus HCs (*n* = 192 for STEMI patients and 100 for HCs). Abbreviations: TLR2: Toll-like receptor 2; TLR4: Toll-like receptor 4; FCGR3B: Fc fragment of IgG receptor IIIb; CAMP: cathelicidin antimicrobial peptide; MMP9: matrix metallopeptidase 9; GZMA: granzyme A; miR-146a: microRNA-146a; miR-146b: microRNA-146b; MACEs: major adverse cardiovascular events; STEMI: ST-segment elevation myocardial infarction; HCs: healthy controls; T4h: 4 hours after the onset of chest pain; IP: immediate postoperative; T24h: 24 hours after the onset of chest pain; T72h: 72 hours after the onset of chest pain; T1W: 1 week after the onset of chest pain.

As shown in Figure 5(a), the expression of S100A12 increased in STEMI patients more than in healthy controls (*P* < 0.05). Meanwhile, there was no difference in the expression of TLR2 (Toll-like receptor 2), TLR4 (Toll-like receptor 4), FCGR3B (Fc fragment of IgG receptor IIIb), CAMP (cathelicidin antimicrobial peptide), MMP9 (matrix metallopeptidase 9), and GZMA (granzyme A) between STEMI patients and the control group. A total of 2 validated miRNA–mRNA relationship pairs (S100A12-miR-146a and S100A12-miR-146b) were predicted by the miRTarBase database. The expression of miRNA-146a was higher in STEMI patients than in healthy controls, while there was no difference in the expression of miRNA-146b (Figure 5(a)). The expression of plasma miR-146a in patients at various times after STEMI onset was compared with that of control subjects (Figure 5(b)).

### 3.5. Baseline Characteristics

At the beginning of the study, 356 STEMI patients were recruited; although, 192 of them were excluded after being removed from follow-up or having died from other illnesses.  Table 1 lists the baseline characteristics of the subjects and comparisons between healthy controls (*n* = 100) and STEMI patients (*n* = 192). WBC, BMI, hs-CRP, LDH, CK, CKMB, hsTn T, and NT-proBNP were significantly higher in STEMI patients than in healthy controls, and the differences were statistically significant (*P* < 0.05). Serological tests showed that there were no significant differences in hemoglobin, LDL, total cholesterol, triglyceride, and LP(a) between the STEMI and control groups (*P* > 0.05). In addition, there were no significant differences in the prevalence of hypertension, DM, and smoking (*P* > 0.05).

### 3.6. Prognosis and Independent Prognostic Factors

**Figure 6:**
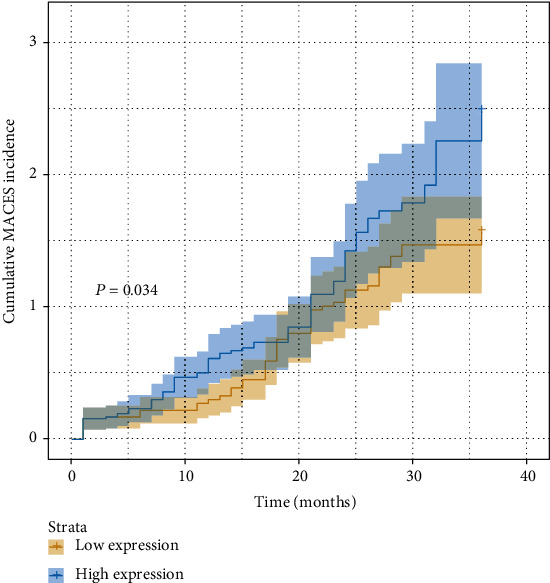
Cumulative incidence of MACEs in STEMI patients. Abbreviations: MACEs: major adverse cardiovascular events; STEMI: ST-segment elevation myocardial infarction.

Univariate analysis was performed on the collected research indicators, and multivariate Cox regression analysis was performed on the statistically significant variables identified by univariate analysis between the two groups. Hazard ratios (HR) and *P* values are shown in Table 2, indicating that age (*P* = 0.005), hypertension (*P* = 0.026), DM (*P* < 0.001), WBC (*P* = 0.002), and miR-146a (*P* = 0.017) were independently related to the outcomes of STEMI patients. STEMI patients with the high expression of miR-146a had a significantly higher probability of experiencing MACEs (*P* = 0.034) compared to those without the high expression of miR-146a, even after eliminating the influence of other risk factors such as gender and age (Figure 6).

### 3.7. Predictive Nomogram for the Probability of MACEs in STEMI Patients

**Figure 7:**
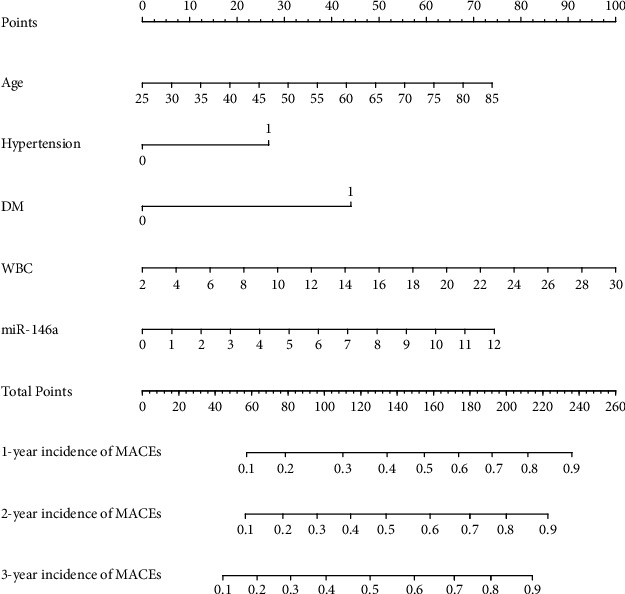
Nomogram based on a stepwise Cox regression model to predict the outcomes of STEMI patients. Abbreviations: MACEs: major adverse cardiovascular events; STEMI: ST-segment elevation myocardial infarction.

A nomogram was generated to predict the 1-, 2-, and 3-year probabilities of MACEs based on the statistically significant variables from the multivariate stepwise Cox regression model (Figure 7). First, the scores of the factors in the nomogram are summed to obtain total points, and then a vertical line is drawn from the total points scale to the probability scale to obtain the 1-, 2-, and 3-year of probabilities of a MACE.

### 3.8. Performance of the Nomogram

**Figure 8:**
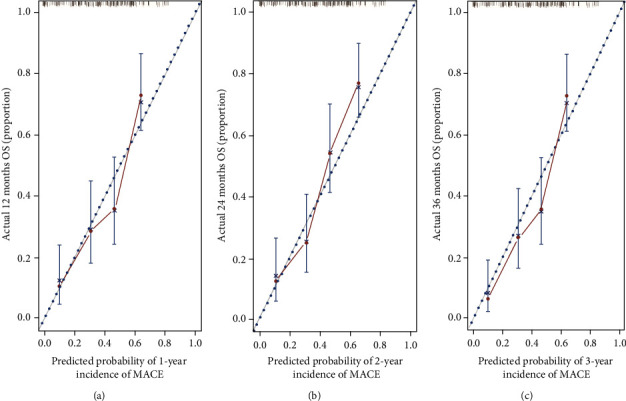
The calibration curve of the incidence of MACEs after 1, 2, and 3 years for STEMI patients. The nomogram-predicted probability of MACEs is plotted on the *x*-axis, and the actual probability of MACEs is plotted on the *y*-axis. Abbreviations: MACEs: major adverse cardiovascular events; STEMI: ST-segment elevation myocardial infarction.

The C-index of the nomogram was 0.685 (95% CI, 0.593–0.752), indicating good discrimination of the nomogram. In addition, the calibration curve indicated similar predicted and actual probabilities for a MACE after 1, 2, or 3 years (Figures 8(a)–8(c)).

## 4. Discussion

STEMI is a major cause of death worldwide and is closely related to abnormal metabolism of endogenous substances and inflammation. The occurrence of STEMI caused by inflammation has been regarded as important for diagnosis, treatment, and prognosis (23, 24). In addition, the possibility of a systemic inflammatory response following acute myocardial infarction cannot be ignored. In the present study, we applied bioinformatic methods to identify 239 DEGs between the STEMI and control groups in the GSE60993 dataset using the *R* language. The hub genes in the PPI network were verified using RT-qPCR. We observed that the expression of S100A12 was significantly upregulated in STEMI samples compared with that in control samples. S100A12 is a calcium-binding protein belonging to the S100 family which exerts significant effects on upregulation of intracytoplasmic Ca^2+^, adhesion of vascular endothelial cells, induction of proinflammatory cytokine generation, and promotion of smooth muscle cell migration (25). S100A12-mediated osteoblastic genes promote remodeling of atherosclerotic plaques and vascular calcification (26), and the expression of S100A12 in the aorta of atherosclerotic rats is higher than in normal tissues (27). Cox proportional hazard analysis showed that S100A12 is an independent factor for predicting the risk of MACEs (28, 29). In addition, S100A12 may serve as a marker of coronary plaque instability and may have therapeutic implications for treatment of acute coronary syndrome (ACS) (30).

Furthermore, information from the miRTarbase database showed that miR-146a can bind to S100A12. We verified that the miR-146a expression was significantly greater in STEMI samples than in control samples (all *P* < 0.05). Binding of miRNA to related proteins protects it from RNase degradation, which can account for stable miRNA levels in blood. Yehuda et al. used well-designed meta-analysis to identify miR-133a as an early biomarker for acute myocardial infarction (31). Such diagnostic miRNA markers of acute coronary syndrome are regularly being discovered, but dependable long-term prognostic markers have yet to be identified. MiR-146a has been shown to be significantly expressed in atherosclerotic arteries (32). There is also evidence that miR-146a is expressed not only in endothelial cells and smooth muscle cells but also in cardiomyocytes (33). Etiologies of STEMI include rupture of local vulnerable plaques and cardiomyocyte necrosis. Our preoperative comparisons of miR-146a between groups are consistent with these results, but the level of miR-146a decreased in the immediate postoperative period. We speculated that successful reperfusion of cardiomyocytes bordering necrotic regions and medications used after admission was the likely causes of this observation. The expression of miR-146a increased gradually during the week after surgery. Baldi et al. detected high-grade apoptosis at sites of infarction at later times following STEMI, which resulted in progressive late left ventricular dysfunction (34). Myocardial necrosis is usually clinically manifested by chest pain symptoms, ECG changes, and myocardial necrosis biomarkers. However, myocardial apoptosis is aphonic. Of course, whether miR-146a is indicative of myocardial apoptosis and postinfarct myocardial remodeling requires further study. He et al. demonstrated that suppression of miR-146a reduces cardiac dysfunction and cardiac remodeling in heart failure rats (35). In addition, miR-146a has been shown to have a role in the development of inflammation and fibrosis. Left ventricular remodeling may be associated with excessive inflammation and fibrosis (36). Therefore, we hypothesized that increased miR-146a levels may contribute to the risk of a MACE after STEMI. Our data show that STEMI patients with high miR-146a levels had a higher risk of MACEs than those with low miR-146a levels during the 3-year follow-up period. It is worth noting that heparin has a negative effect on the accuracy of qRT-PCR (37). Li et al. observed that heparin inhibits miRNA amplification by ∼4 cycles (38). Schulte et al. showed that heparin reduces the detectability of miR-39, which could be reversed by heparinase treatment (39). It is therefore important to bear in mind heparinization associated with PCI (percutaneous coronary intervention) surgery.

Age, hypertension, DM, WBC, and miR-146a played a role in our predictive model. Human functions and physiological processes decline with age, which is likely linked with a variety of other illnesses. Furthermore, the coronary arteries of older individuals are often characterized by widespread venereal infections and significant calcification, lowering the likelihood of effective vascular repair (40). Hypertension and DM are traditional risk factors for cardiovascular events (41). Duan et al. showed that the incidence of MACEs, cardiovascular disease mortality, and stroke is higher in rural China for people with prehypertension (42). Okkonen also found that the most important risk factor for MACE after ACS is diabetes patients in a Finnish myocardial infarction register (43). Long-term prognosis of ACS is affected by inflammation. In addition, white blood cells play an important role in the release of inflammatory cytokines (44). Recent studies have found that MACEs are more likely if the WBC–platelet volume ratio is elevated in individuals with non-ST elevation acute coronary syndrome (NSTEMI) (45). In addition, MACEs in patients with AMI have been linked to increased risk of having certain WBC subtypes (46). Therefore, the risk of MACEs in hospitalized STEMI patients can be lowered significantly by reducing blood pressure, blood glucose, and WBC levels.

## 5. Conclusion

The incidence of MACEs in STEMI patients with the high miR-146a expression was substantially greater than in the low expression group. Therefore, miR-146a might function in the pathogenesis of STEMI by targeting S100A12, which may exert its role via an inflammatory response and serve as a biomarker for clinical diagnosis and adverse prognosis of STEMI. In addition, our research provides a reliable and feasible methodology to evaluate the likelihood of MACEs within three years of STEMI.

## 6. Limitations to the Study

Firstly, it is challenging to take into account critical variables such as locale and race. Secondly, there is a plausible association between miR-146a levels and heparinization. The effect of heparin on detection of miR-146a cannot be excluded because heparinization is required for PCI. Finally, further studies are required to establish the mechanism by which miR-146a functions.

## Figures and Tables

**Table 1:** Baseline characteristics of all study participants.

Variable	Control (*n* = 100)	STEMI (*n* = 192)	*P* value
Age, years	61.65 ± 12.91	62.27 ± 12.24	0.689
Gender (*n*, %)			0.315
Male	64 (64.00%)	134 (69.79%)	
Female	36 (36.00%)	58 (30.21%)	
Hypertension (*n*, %)			0.346
No	66 (66.00%)	137 (71.35%)	
Yes	34 (34.00%)	55 (28.65%)	
Diabetes mellitus (*n*, %)			0.284
No	82 (82.00%)	147 (76.56%)	
Yes	18 (18.00%)	45 (23.44%)	
Smoke (*n*, %)			0.243
No	82 (82.00%)	146 (76.04%)	
Yes	18 (18.00%)	46 (23.96%)	
BMI, kg/m^2^	23.13 ± 3.64	24.61 ± 2.14	<0.001
WBC, ×10^9^/L	6.08 ± 1.77	11.67 ± 5.09	<0.001
Hemoglobin, g/L	104.64 ± 6.59	101.28 ± 12.22	0.011
hs-CRP, mg/L	8.51 ± 41.54	31.90 ± 23.07	<0.001
HDL-C, mmol/L	1.26 ± 0.26	1.06 ± 0.25	<0.001
LDL-C, mmol/L	2.73 ± 0.75	2.77 ± 0.74	0.67
Total cholesterol, mmol/L	4.58 ± 0.90	4.31 ± 0.84	0.013
Triglyceride, mmol/L	1.36 ± 0.79	1.42 ± 0.63	0.479
LDH, U/L	171.50 ± 39.02	695.38 ± 300.29	<0.001
CK, U/L	92.41 ± 49.19	720.78 ± 199.33	<0.001
CKMB, ng/mL	1.97 ± 1.04	112.55 ± 55.82	<0.001
hsTn T, ng/L	17.22 ± 10.08	783.32 ± 462.74	<0.001
NT-proBNP, pg/mL	830.86 ± 471.41	1801.27 ± 625.33	<0.001
LP(a), mg/L	207.65 ± 162.31	254.23 ± 160.02	0.019

Abbreviations: STEMI: ST-Segment elevation myocardial infarction; WBC: white blood cell; hs-CRP: high-sensitivity C-reactive protein; HDL-C: high-density lipoprotein-cholesterol; LDL: low-density lipoprotein-cholesterol; LDH: lactate dehydrogenase; CK: creatine kinase; CKMB: creatine kinase MB; hsTn T: hypersensitive troponin T; NT-proBNP: N-terminal probrain natriuretic peptide; Lp(a): lipoprotein-a.

**Table 2:** Univariate and multivariate Cox regression analysis of prognosis in the population.

Variables	Univariate analysis	*P* value	Multivariate analysis	*P* value
	HR (95% CI)		HR (95% CI)	
Age	1.366 (1.070, 1.743)	0.012	1.422 (1.079, 1.824)	0.005
Hypertension	1.408 (1.009, 1.965)	0.041	1.485 (1.049, 2.103)	0.026
DM	1.731 (1.219, 2.456)	0.002	1.922 (1.334, 2.769)	<0.001
WBC	1.5409 (1.173, 2.003)	0.002	1.519 (1.155, 1.995)	0.002
miR-146a	1.285 (1.047, 1.584)	0.017	1.329 (1.060, 1.664)	0.01

Abbreviations: DM: diabetes mellitus; WBC: white blood cell.

## Data Availability

The datasets used in this research are accessible on a reasonable request from the corresponding author.

## Supplementary Materials

Supplementary 1 Supplementary Table S1: characteristics of healthy controls (HCs group) and ST-segment elevation myocardial infarction patients (STEMI group).

Supplementary 2 Supplementary Table S2: primers used in this study.

Supplementary 3 Supplementary File S3: inclusion and exclusion criteria of participants.

Supplementary 4 Supplementary File S4: informed consent and questionnaire from the Affiliated Hospital of Xuzhou Medical University.

Supplementary 5 Supplementary Table S5: the DEGs screened between the STEMI control group.

Supplementary 6 Supplementary Table S6: Gene Ontology analysis of the DEGs.

Supplementary 7 Supplementary Table S7: Kyoto Encyclopedia of Genes and Genomes pathway analysis of the DEGs.
