# Supplementary material for: MicroRNA-146a Serves as a Biomarker for Adverse Prognosis of ST-Segment Elevation Myocardial Infarction
Source: Cardiovasc Ther. 2021 Oct 25;2021:2923441. doi: 10.1155/2021/2923441 (PMC8561321; doi:10.1155/2021/2923441)
Supplement: Supplementary 1 — Supplementary Table S1: characteristics of healthy controls (HCs group) and ST-segment elevation myocardial infarction patients (STEMI group). [file 2923441.f1.docx]

| Supplementary Table S1. Characteristics of healthy controls (HCs group) and ST-segment elevation myocardial infarction patients (STEMI group). | | | |
| --- | --- | --- | --- |
| Initial validation |  |  |  |
| Variables | Control (n=4) | AMI (n=4) | *P*-value |
| Age, years | 60±7.439 | 60±6.446 | 0.914 |
| Sex (n, %) |  |  |  |
| Male | 2(50%) | 2(50%) | 0.757 |
| Female | 2(50%) | 2(50%) |  |
| Hypertension (n, %) |  |  |  |
| Yes | 1(25%) | 1(25%) | 0.786 |
| No | 3(75%) | 3(75%) |  |
| Diabetes mellitus (n, %) |  |  | 0.786 |
| Yes | 1(25%) | 1(25%) |  |
| No | 3(75%) | 3(75%) |  |
| Smoking (n, %) |  |  |  |
| Yes | 3(75%) | 1(25%) | 0.243 |
| No | 1(25%) | 3(75%) |  |
| BMI, kg/m^2^ | 28.025±2.145 | 30.725±2.575 | 0.599 |
| WBC, 10^9^/L | 5.500±2.064 | 9.000±4.075 | 0.073 |
| Hemoglobin, g/L | 133.000±12.623 | 144.500±11.504 | 0.227 |
| Hs-CRP, 10^9^/L | 289.000±123.852 | 194.000±68.503 | 0.358 |
| TG, mmol/L | 1.186±0.196 | 1.928±1.694 | 0.055 |
| TC, mmol/L | 3.190±0.869 | 5.330±1.150 | 0.817 |
| HDL, mmol/L | 1.100±0.089 | 1.085±0.148 | 0.466 |
| LDL, mmol/L | 2.468±0.810 | 3.460±0.906 | 0.418 |
| Lp(a), mg/L | 167.750±107.140 | 280.500±230.701 | 0.423 |
| LDH, U/L | 172.500±40.402 | 576.000±214.465 | 0.09 |
| CK, U/L | 79.000±13.976 | 318.250±222.187 | 0.12 |
| CKMB, ng/ml | 2.050±0.540 | 10.350±8.517 | 0.146 |
| hsTn T, ng/L | 4.810±2.150 | 416.000±137.247 | 0.009 |
| NT-proBNP, pg/ml | 211.500±50.428 | 398.250±178.849 | 0.125 |
| Lp(a), mg/L | 167.750±107.140 | 280.500±230.701 | 0.423 |

Abbreviations: DM, Diabetes mellitus; BMI, body mass index; WBC, white blood cell; Hs-CRP, high-sensitivity C-reactive protein; TG, triglyceride; TC, Total cholesterol; HDL, High density lipoprotein; LDL, Low Density Lipoprotein; Lp(a), Lipoprotein-a; LDH, lactate dehydrogenase; CK, creatine kinase; CKMB, creatine kinase MB; hsTn T, Hypersensitive troponin T; NT-proBNP, N-terminal pro-brain natriuretic peptide;
