# Supplementary material for: MicroRNA-146a Serves as a Biomarker for Adverse Prognosis of ST-Segment Elevation Myocardial Infarction
Source: Cardiovasc Ther. 2021 Oct 25;2021:2923441. doi: 10.1155/2021/2923441 (PMC8561321; doi:10.1155/2021/2923441)
Supplement: Supplementary 2 — Supplementary Table S2: primers used in this study. [file 2923441.f2.docx]

| Supplementary Table S2. Primers used in this study. | | |
| --- | --- | --- |
| Gene | Forward Primer | Reverse Primer |
| TLR2 | ATCCTCCAATCAGGCTTCTCT | GGACAGGTCAAGGCTTTTTACA |
| TLR4 | CCTCGGCGGCAACTTCATAA | AGAGCGGATCTGGTTGTACTG |
| FCGR3B | GCTCTGCTACTTCTAGTTTCAGC | CTCCCTGGCACTTCAGAGTC |
| CAMP | GAACCGCAGTATCATGCTGG | TCCTTGAATTAAGCCGTTCATCA |
| MMP9 | TGTACCGCTATGGTTACACTCG | GGCAGGGACAGTTGCTTCT |
| GZMA | TCTCTCTCAGTTGTCGTTTCTCT | GCAGTCAACACCCAGTCTTTTG |
| S100A12 | AGCATCTGGAGGGAATTGTCA | GCAATGGCTACCAGGGATATGAA |
| miR-146a | GTGCAGGGTCCGAGGT | CGGCGGTGAGAACTGAATTCC |
| miR-146b | TGACCCATCCTGGGCCTCAA | CCAGTGGGCAAGATGTGGGCC |
| Human-18S | CAGCCACCCGAGATTGAGCA | TAGTAGCGACGGGCGGTGTG |
