# Supplementary material for: MicroRNA-146a Serves as a Biomarker for Adverse Prognosis of ST-Segment Elevation Myocardial Infarction
Source: Cardiovasc Ther. 2021 Oct 25;2021:2923441. doi: 10.1155/2021/2923441 (PMC8561321; doi:10.1155/2021/2923441)
Supplement: Supplementary 3 — Supplementary File S3: inclusion and exclusion criteria of participants. [file 2923441.f3.docx]

**Supplementary File S3. Inclusion and exclusion criteria of participants.**

**Inclusion criteria** **of patients:**

(1) Patients with obvious clinical symptoms of myocardial ischemia, such as chest pain, chest tightness or shortness of breath, whose attack duration exceeds 30 minutes and cannot be completely relieved by nitroglycerin.

(2) ECG showed at least two consecutive anterior leads or at least two adjacent limb leads, ST segment elevation of 0.1mV, or new (possibly new) left bundle branch block, with a dynamic evolution of myocardial ischemia.

(3) The elevation of myocardial enzyme exceeded the upper 99th percentile of the reference value and showed a dynamic evolution.

(4) Coronary angiography confirmed target vessel stenosis.

**Exclusion criteria of acute myocardial infarction:**

Individuals with a history of congenital heart disease, rheumatic heart disease, viral myocarditis, chronic obstructive pulmonary disease, peripheral vascular diseases, recent history of infection or trauma, cancer, autoimmune disorders, abnormal renal/liver function, hemopathy or thyroid dysfunction were excluded.

**Healthy controls selection criteria:**

HCs matched by ethnicity (Han Chinese), age, and sex were also recruited. All participants were healthy, and none of them had a history of ischemic stroke, valvular heart disease, congenital heart disease, cardiomyopathy and chronic heart failure as determined by history-taking, questionnaires, and clinical examination.
