# Supplementary material for: MicroRNA-146a Serves as a Biomarker for Adverse Prognosis of ST-Segment Elevation Myocardial Infarction
Source: Cardiovasc Ther. 2021 Oct 25;2021:2923441. doi: 10.1155/2021/2923441 (PMC8561321; doi:10.1155/2021/2923441)
Supplement: Supplementary 4 — Supplementary File S4: informed consent and questionnaire from the Affiliated Hospital of Xuzhou Medical University. [file 2923441.f4.pdf]

Informed consent and questionnaire from the Affiliated Hospital of Xuzhou Medical University

Dear Participants:

We warmly invite you to participate in a research project “MicroRNA-146a Serve as Biomarkers for Adverse Prognosis of ST-segment Elevation Myocardial Infarction” approved by the ethics committee of the Affiliated Hospital of Xuzhou Medical University. This study will be conducted in the Affiliated Hospital of Xuzhou Medical University, and it is estimated that 400 subjects will voluntarily participate.

You have abundant rights to decide whether participate our study. Even if you have signed your consent to participate, you can change your decision at any stage of the study to abandon participation. No reason is needed for withdrawing from the study and we fully respect your autonomy.

Why was this study conducted?

ST-segment elevation myocardial infarction (STEMI) is a serious type of coronary heart disease and one of the diseases with the highest death and disability. The current protocol for detecting a myocardial infarction uses hs-Tn T, which is a high-sensitivity troponin T. However, there is a lack of prognostic biomarkers for STEMI. Therefore, in order to better judge the prognosis of STEMI, new biomarkers need to be explored in the blood.

MicroRNAs (miRNAs) are a class of small non-coding RNAs that exist not only in prokaryotic cells, but also in large numbers in many eukaryotic organisms, and regulate 30% of genes in eukaryotic organisms after transcription. At present, more and more studies believe that miRNAs may promote

the occurrence and development of STEMI by regulating various potential signaling pathways. Currently, a number of studies at home and abroad have confirmed that miRNAs are involved in coronary atherosclerosis, acute myocardial infarction, myocardial fibrosis after infarction, and cardiac remodeling, etc. In addition, miRNA plays an important role in the physiological and pathological processes of the heart, such as cardiac development, arrhythmia, heart failure, cardiac hypertrophy, myocardial injury, etc. The change of miRNAs expression level is closely related to disease or injury. Hence, the level of miRNAs in the blood circulation can be used as biomarkers to evaluate and monitor pathophysiologic states.

Regarding STEMI, miRNAs can be used not only as a biological marker but also as a molecular targeted drug for the treatment. With the wide application of RNA sequencing technology and rapid development of biophysical techniques, the role of miRNAs in gene regulation and disease occurrence is attracting attention. The prognostic potential of miR-146a was forecasted by analyzing microarray data. To further confirm the potential of miR-146a as a prognostic biomarker, we will exame miR-146a expression in patients who meet the inclusion criteria.

Who should not be included in the study?

If you are: I) patients with previous history of heart valve disease, congenital heart disease, cardiomyopathy, chronic heart failure, II) patients with previous stroke, familial hypercholesterolemia, severe hepatorenal pulmonary and thyroid disease, neoplasm, sepsis and patients with familial and hereditary diseases, III) pregnant, complicated by local or systemic infection; IV) mental illness, alcohol addiction, drug abusers.

What needs to be done if enrolled in the study?

If you are a patient with STEMI and willing to participate in this study, you will be required to

provide 5 ml of cubital venous blood immediately (within 4 hours), immediately post-surgery, 24 hours, 48 hours after surgery to answer and fill in the following questions.

If you are a healthy volunteer and willing to participate in this study, you will need to provide 5 ml of cubital venous blood and answer and fill in the following questions.

1. Gender：□Male □Female
2. Age_______（year）
3. Height_______（cm）
4. Weight_______（kg）
5. History of surgery：□No □Yes（______years ago, term of operation：_________）

   Receiving blood products：□No □Yes
6. Smoke：□No □Yes（______years, ______branches/day，forbid smoking for approximately ______year）
7. Drink：□No □Yes（Category □Liquor and spirits □Wine □Beer，____year, ____g/day，frobid drinking for approximately ____days）
8. Physical activity：□Normal □Restricted
9. Hypertension：□No □Unclear □Yes（Highest___/___mmHg, Lowest____/___mmHg; Feel dizzy when blood pressure up to ______mmHg，Normal blood pressure _______ mmHg）
10. Diabetes mellitus：□No □Unclear □Yes（□Drug □Insulin）
11. History of coronary heart disease：□No □Yes
12. Dietary：□Normal □Over □Less □Unable to eat
13. History of gastric or duodenal ulcers：□No □Unclear □Yes
14. History of drug allergy：□No □Yes（Name___________）

15. History of food allergy：□No □Yes（Name______________）

16. The recent medication：□No □Yes（Name_____________）

17. Menstruation：□Menstrual period □Non-menstrual period

18. Relatives (blood relationship) related diseases:-without/with _____________________ (name)_____________________）

What are the risks of participating in the study?

Some adverse reactions occur with venous blood sampling: 1. Local hematoma and stasis 2. Needle eye bleeding 3. Skin allergy 4. Phlebitis 5. Subcutaneous vascular injury 6. Fainting.

What are the benefits of participating in the study?

This study will screen out some novel candidate genes and miRNAs that may serve as prognosis biomarkers of STEMI by mediating aspects of inflammatory response, plaque and thrombosis, angiogenesis, myocardial remodeling, myocardial fibrosis, cardiomyocyte proliferation, apoptosis, and so on, expecting that through follow-up studies, they may provide new biological targets and research directions for STEMI.

Is there a fee to pay for participating in the study?

You are not required to pay for this, and if a study related adverse reaction occurs, we will take steps to prevent it.

Do I have to take part in the study?

Participation in this study is completely voluntary and you may refuse to participate in the study, or withdraw from the study at any time during the course of the study, which will not affect your treatment by your doctor. If you decide to withdraw from this study, please contact your doctor and you may be asked for the relevant tests, which are beneficial for protecting your health.

If you believe that study damage has occurred, please contact your study doctor at the following contact number: 13852438611.

If you feel your interests are compromised, please contact the hospital ethics committee at: 0516-85806993.

Subject statement: I have read the above introduction to this study, being well informed of the risks and benefits that may result from participation in this study.

I volunteered to participate in this study. I agree □ decline □ other studies than this study utilize my medical records and specimens for pathologic examination.

Subject's signature: Date:

Subject's contact phone: Mobile phone:

Doctor states: I confirm that details of this study have been explained to the patient, in particular the risks and benefits that may result from participating in this study.

Physician's signature: Date:

Physician's working phone: Mobile phone:

Clinical trial ethics committee of Affiliated Hospital of Xuzhou Medical University contact number: 0516-85802291.
