# Supplementary material for: MicroRNA-146a Serves as a Biomarker for Adverse Prognosis of ST-Segment Elevation Myocardial Infarction
Source: Cardiovasc Ther. 2021 Oct 25;2021:2923441. doi: 10.1155/2021/2923441 (PMC8561321; doi:10.1155/2021/2923441)
Supplement: Supplementary 5 — Supplementary Table S5: the DEGs screened between the STEMI control group. [file 2923441.f5.docx]

| Supplementary Table S5. The DEGs screened between the STEMI-Control group | | | |
| --- | --- | --- | --- |
| Gene names | logFC | *P*-value | Change |
| MMP9 | 3.165649429 | 4.08E-05 | UP |
| FCGR3B | 2.308436286 | 0.03897604 | UP |
| ARG1 | 2.297344586 | 0.000155057 | UP |
| ORM1 | 2.290833114 | 0.003570807 | UP |
| IL18R1 | 2.029458957 | 7.07E-07 | UP |
| IL1R2 | 1.888213571 | 0.000254515 | UP |
| PGLYRP1 | 1.886529 | 0.000442282 | UP |
| GPR97 | 1.884963514 | 1.60E-05 | UP |
| MOSC1 | 1.879211 | 1.70E-05 | UP |
| ECHDC3 | 1.875256829 | 0.000347934 | UP |
| QPCT | 1.871023786 | 2.90E-07 | UP |
| CA4 | 1.852039986 | 0.000564633 | UP |
| VNN3 | 1.850013571 | 1.42E-06 | UP |
| PADI4 | 1.845429 | 6.48E-05 | UP |
| IRS2 | 1.788734814 | 7.63E-07 | UP |
| MCEMP1 | 1.753685 | 2.50E-06 | UP |
| FOLR3 | 1.742984571 | 0.006626782 | UP |
| CAMP | 1.731777 | 0.002288101 | UP |
| MANSC1 | 1.690506 | 5.24E-08 | UP |
| LOC653117 | 1.681150686 | 3.45E-07 | UP |
| CYP4F3 | 1.679492786 | 1.50E-06 | UP |
| LOC642103 | 1.678735643 | 5.76E-08 | UP |
| CLEC4E | 1.675906971 | 9.55E-08 | UP |
| CRISPLD2 | 1.647243357 | 6.84E-05 | UP |
| FOS | 1.638083686 | 2.04E-06 | UP |
| ACSL1 | 1.612098286 | 1.01E-07 | UP |
| TGM3 | 1.610271629 | 5.68E-05 | UP |
| SLC26A8 | 1.609947371 | 1.40E-06 | UP |
| PHC2 | 1.609931857 | 9.58E-05 | UP |
| UTS2 | 1.589751386 | 0.030670983 | UP |
| CLEC4D | 1.563777829 | 0.000354167 | UP |
| S100A12 | 1.539724071 | 7.40E-05 | UP |
| ROPN1L | 1.532129714 | 0.000556472 | UP |
| PBEF1 | 1.530832571 | 9.52E-06 | UP |
| PANX2 | 1.521115571 | 7.03E-05 | UP |
| DUSP1 | 1.517304643 | 3.73E-05 | UP |
| MME | 1.509962571 | 3.17E-05 | UP |
| LOC441268 | 1.4716141 | 0.001504447 | UP |
| HMGB2 | 1.450047786 | 5.83E-06 | UP |
| HLA-DRB1 | 1.446802129 | 0.041456794 | UP |
| RGS2 | 1.444582714 | 2.80E-06 | UP |
| IRAK3 | 1.431971357 | 9.05E-07 | UP |
| LOC642342 | 1.429627243 | 0.001694871 | UP |
| DYSF | 1.422904571 | 0.001696959 | UP |
| FKBP5 | 1.411722 | 1.14E-05 | UP |
| PYGL | 1.394143714 | 1.23E-05 | UP |
| HLA-C | 1.382410357 | 0.01318464 | UP |
| CDA | 1.369223714 | 0.000824458 | UP |
| NOV | 1.3632888 | 6.15E-07 | UP |
| USP10 | 1.361717143 | 0.000135623 | UP |
| IFRD1 | 1.348033043 | 0.000172254 | UP |
| FPRL1 | 1.342063429 | 9.70E-05 | UP |
| LTB4R | 1.3409904 | 4.62E-05 | UP |
| HIST1H4H | 1.332319843 | 4.73E-05 | UP |
| CR1 | 1.331844614 | 9.99E-08 | UP |
| CEBPD | 1.331271714 | 5.97E-09 | UP |
| SIPA1L2 | 1.330500014 | 4.50E-05 | UP |
| LOC652616 | 1.330148429 | 0.000425697 | UP |
| FAM101B | 1.323868429 | 0.007830587 | UP |
| KCNJ15 | 1.320960714 | 2.64E-07 | UP |
| SLC11A1 | 1.319787786 | 8.09E-05 | UP |
| FBXL13 | 1.3053402 | 0.002101494 | UP |
| TLR4 | 1.304876643 | 2.52E-06 | UP |
| HAL | 1.300505643 | 3.85E-07 | UP |
| PROK2 | 1.294448286 | 0.000644629 | UP |
| SLC37A3 | 1.294135043 | 0.001259625 | UP |
| HECW2 | 1.292956371 | 4.85E-06 | UP |
| MGAM | 1.292564643 | 7.42E-08 | UP |
| TCN1 | 1.287116743 | 0.001572419 | UP |
| PFKFB3 | 1.285957214 | 6.05E-06 | UP |
| GCA | 1.285281143 | 0.000518322 | UP |
| FRAT1 | 1.279713357 | 3.40E-07 | UP |
| CEACAM4 | 1.279254657 | 1.93E-05 | UP |
| CD55 | 1.275806743 | 0.000231932 | UP |
| STX3A | 1.269443714 | 7.01E-06 | UP |
| CKAP4 | 1.264123143 | 3.87E-05 | UP |
| MEGF9 | 1.263839657 | 1.25E-06 | UP |
| PELI1 | 1.2560165 | 0.001561124 | UP |
| TPST1 | 1.248884886 | 0.003853497 | UP |
| NALP12 | 1.237926857 | 2.49E-05 | UP |
| STK17B | 1.236324286 | 1.57E-05 | UP |
| B4GALT5 | 1.235549286 | 8.29E-06 | UP |
| SIGLEC5 | 1.235165929 | 0.00075358 | UP |
| HIP1 | 1.230406743 | 2.73E-05 | UP |
| NDST1 | 1.228377657 | 0.00021045 | UP |
| ABHD5 | 1.2256478 | 5.68E-05 | UP |
| LMNB1 | 1.223517429 | 8.02E-06 | UP |
| CECR6 | 1.216878086 | 0.001363409 | UP |
| SLC22A15 | 1.213409614 | 2.18E-06 | UP |
| ALOX5AP | 1.2078205 | 8.47E-08 | UP |
| GAB2 | 1.204313786 | 6.66E-08 | UP |
| LILRA2 | 1.202328643 | 6.06E-07 | UP |
| RGS18 | 1.199457714 | 0.015015079 | UP |
| NCF1 | 1.194178643 | 0.000568726 | UP |
| CPD | 1.193981571 | 1.75E-05 | UP |
| HMFN0839 | 1.184920286 | 3.47E-06 | UP |
| KIAA0319L | 1.181230629 | 0.000595533 | UP |
| LOC654053 | 1.179693443 | 7.81E-05 | UP |
| NFIL3 | 1.179117657 | 2.07E-06 | UP |
| TP53I11 | 1.178018871 | 0.002118318 | UP |
| GPR177 | 1.177755286 | 0.000895143 | UP |
| IL18RAP | 1.175675714 | 0.000617066 | UP |
| NCF4 | 1.175558429 | 9.03E-05 | UP |
| LOC283547 | 1.172878971 | 0.000588833 | UP |
| RBP7 | 1.166573429 | 2.45E-05 | UP |
| SAP30 | 1.163012843 | 0.000963517 | UP |
| CREB5 | 1.162304571 | 0.000839702 | UP |
| SLC40A1 | 1.160253857 | 0.001708246 | UP |
| ANPEP | 1.159952786 | 0.001211037 | UP |
| CHST13 | 1.158938857 | 0.000275824 | UP |
| CSTA | 1.153227271 | 8.26E-07 | UP |
| IL8 | 1.1521491 | 0.02438717 | UP |
| GNG10 | 1.149998157 | 0.020091965 | UP |
| LOC349114 | 1.148356857 | 0.00067767 | UP |
| BMX | 1.147770043 | 9.64E-05 | UP |
| BST1 | 1.145094471 | 3.69E-07 | UP |
| FLJ22662 | 1.142143286 | 5.51E-06 | UP |
| EMR3 | 1.13916 | 0.000210523 | UP |
| IL8RA | 1.137087786 | 0.000904611 | UP |
| RRAGD | 1.1366215 | 5.71E-05 | UP |
| TREM1 | 1.134847557 | 0.000370525 | UP |
| LILRA3 | 1.134365943 | 0.013133324 | UP |
| NCF1C | 1.132326 | 0.000528825 | UP |
| HIST2H2BE | 1.130823343 | 0.001128651 | UP |
| KIAA1026 | 1.127632471 | 0.002562204 | UP |
| ORF1-FL49 | 1.125046929 | 0.001769886 | UP |
| PDLIM7 | 1.123739829 | 0.002039338 | UP |
| ST6GALNAC2 | 1.122392329 | 0.000201333 | UP |
| NCF1B | 1.119423957 | 0.001629359 | UP |
| LBR | 1.118305214 | 4.79E-05 | UP |
| NRBF2 | 1.116368 | 0.000163812 | UP |
| RTN3 | 1.108861429 | 3.73E-07 | UP |
| MMP25 | 1.108701 | 0.000385273 | UP |
| KRT23 | 1.1084516 | 0.003192124 | UP |
| LOC399744 | 1.107816143 | 0.001951525 | UP |
| C1QR1 | 1.107604143 | 0.000200776 | UP |
| LENG4 | 1.106966143 | 0.000968079 | UP |
| PTGS2 | 1.105157457 | 0.006903226 | UP |
| C5AR1 | 1.1022515 | 5.91E-05 | UP |
| FKBP9L | 1.099783229 | 9.92E-06 | UP |
| CDK5R1 | 1.097194971 | 0.00100204 | UP |
| TM6SF1 | 1.092015757 | 1.49E-05 | UP |
| REPS2 | 1.091270429 | 0.001240696 | UP |
| C13orf18 | 1.083855 | 8.15E-05 | UP |
| Rgr | 1.080275143 | 0.000782523 | UP |
| C20orf3 | 1.075533643 | 6.46E-06 | UP |
| C19orf35 | 1.071196443 | 0.000219158 | UP |
| LOC652878 | 1.069386 | 7.45E-05 | UP |
| TMEM91 | 1.069113 | 0.000119024 | UP |
| SLC22A4 | 1.068524571 | 0.001997915 | UP |
| FAM126B | 1.067517286 | 0.001068403 | UP |
| CXCL16 | 1.066283571 | 8.37E-05 | UP |
| OPLAH | 1.063918986 | 0.000285501 | UP |
| EGFL5 | 1.0621257 | 0.000944043 | UP |
| RNF149 | 1.056871571 | 0.000136805 | UP |
| AQP9 | 1.054557429 | 6.00E-05 | UP |
| TMEM71 | 1.049845857 | 0.001867519 | UP |
| LRG1 | 1.048835786 | 0.000530795 | UP |
| CMTM2 | 1.047915143 | 0.00151068 | UP |
| SDCBP | 1.046379571 | 0.000660126 | UP |
| CD46 | 1.043645857 | 0.011566742 | UP |
| ZDHHC18 | 1.039892857 | 0.000219846 | UP |
| CITED4 | 1.038746957 | 0.005266538 | UP |
| C1orf183 | 1.038499186 | 0.006570976 | UP |
| FPR1 | 1.036980714 | 0.000604343 | UP |
| VMD2 | 1.035335571 | 9.45E-05 | UP |
| VNN2 | 1.035044 | 1.03E-07 | UP |
| TLR2 | 1.0347469 | 0.001105525 | UP |
| ACOX1 | 1.0336082 | 9.59E-07 | UP |
| OLFM4 | 1.032631614 | 0.010068646 | UP |
| MXD1 | 1.029168 | 7.05E-05 | UP |
| BPI | 1.022956914 | 7.69E-05 | UP |
| C12orf35 | 1.022402571 | 0.002089977 | UP |
| HIST2H2AA3 | 1.022211357 | 0.00046462 | UP |
| RAB27A | 1.017787243 | 0.000378746 | UP |
| NQO2 | 1.016163186 | 0.002908416 | UP |
| TSEN34 | 1.014061071 | 6.74E-06 | UP |
| GALNAC4S-6ST | 1.013780429 | 3.34E-07 | UP |
| BCL3 | 1.012556714 | 2.99E-05 | UP |
| SULF2 | 1.007891571 | 6.37E-06 | UP |
| LOC648394 | 1.007844929 | 0.000127005 | UP |
| CD58 | 1.007497643 | 0.001255617 | UP |
| SULT1A2 | 1.007243314 | 3.66E-05 | UP |
| HSDL2 | 1.006545629 | 1.60E-05 | UP |
| APAF1 | 1.004895643 | 4.61E-05 | UP |
| ALPL | 1.004441071 | 0.014808941 | UP |
| DIRC2 | 1.003848571 | 1.75E-06 | UP |
| F5 | 1.003586943 | 0.001448355 | UP |
| MAP1LC3A | 1.003034929 | 0.019994316 | UP |
| RP2 | 1.0027468 | 0.003803631 | UP |
| FLJ25084 | 1.002591686 | 0.000691758 | UP |
| PSG11 | 1.001744529 | 0.003645389 | UP |
| PLAUR | 1.0006865 | 7.65E-05 | UP |
| FLT3LG | -1.001065643 | 7.54E-07 | DOWN |
| NKG7 | -1.008397286 | 1.31E-06 | DOWN |
| MGC2463 | -1.008886929 | 5.91E-05 | DOWN |
| LOC642989 | -1.014367786 | 0.001370791 | DOWN |
| LAT | -1.014674143 | 5.19E-06 | DOWN |
| BIN1 | -1.017265286 | 0.0007018 | DOWN |
| CX3CR1 | -1.021037429 | 0.017592666 | DOWN |
| NCALD | -1.024061286 | 3.12E-08 | DOWN |
| ITM2A | -1.027823786 | 4.06E-05 | DOWN |
| GPR56 | -1.031183857 | 0.002530812 | DOWN |
| GIMAP5 | -1.033884214 | 2.96E-06 | DOWN |
| IL2RB | -1.036845286 | 0.000573017 | DOWN |
| ADA | -1.039803643 | 2.92E-07 | DOWN |
| PYHIN1 | -1.042840929 | 6.94E-05 | DOWN |
| BOLA2 | -1.045803786 | 0.001198424 | DOWN |
| GPR114 | -1.048331714 | 5.33E-06 | DOWN |
| RPS26L1 | -1.048392 | 0.0151928 | DOWN |
| CD8A | -1.053810786 | 0.002295695 | DOWN |
| LOC648470 | -1.063605529 | 0.018214917 | DOWN |
| LOC644928 | -1.063962357 | 0.021041639 | DOWN |
| ACSM3 | -1.068560614 | 0.034427385 | DOWN |
| PLEKHF1 | -1.0795665 | 4.06E-07 | DOWN |
| ZAP70 | -1.095300429 | 5.27E-06 | DOWN |
| KSP37 | -1.098081571 | 0.001455424 | DOWN |
| CDC25B | -1.099164 | 2.29E-05 | DOWN |
| SBK1 | -1.099195571 | 1.52E-06 | DOWN |
| CD3D | -1.100249 | 1.84E-05 | DOWN |
| ITGB7 | -1.105348429 | 0.00010562 | DOWN |
| MATK | -1.119449286 | 0.000154086 | DOWN |
| LOC642113 | -1.126862286 | 0.008618721 | DOWN |
| GZMM | -1.148054286 | 0.000155085 | DOWN |
| OLIG2 | -1.167934957 | 0.000639419 | DOWN |
| EDG8 | -1.174595786 | 1.75E-05 | DOWN |
| GZMH | -1.183145929 | 2.82E-05 | DOWN |
| GZMA | -1.206927929 | 0.001554471 | DOWN |
| HLA-DQA1 | -1.211850429 | 0.003582042 | DOWN |
| LOC647450 | -1.213628857 | 0.003022007 | DOWN |
| GNLY | -1.222059857 | 0.001163295 | DOWN |
| CD52 | -1.2352685 | 0.001188673 | DOWN |
| KLRB1 | -1.235579571 | 0.000787751 | DOWN |
| LOC652493 | -1.276362143 | 0.001485881 | DOWN |
| KLRG1 | -1.276917571 | 0.001139862 | DOWN |
| PRSS33 | -1.278338657 | 0.004166868 | DOWN |
| EOMES | -1.327265786 | 1.19E-05 | DOWN |
| CLC | -1.461970357 | 0.000264566 | DOWN |
| GZMK | -1.551611857 | 0.000233429 | DOWN |

Abbreviations: Positive logFC values indicate upregulated expression in STEMI samples compared with in normal samples, whereas negative logFC values represent downregulated expression in STEMI samples compared with in normal samples. DEGs, differentially expressed genes; FC, fold change;
