# Supplementary material for: MicroRNA-146a Serves as a Biomarker for Adverse Prognosis of ST-Segment Elevation Myocardial Infarction
Source: Cardiovasc Ther. 2021 Oct 25;2021:2923441. doi: 10.1155/2021/2923441 (PMC8561321; doi:10.1155/2021/2923441)
Supplement: Supplementary 6 — Supplementary Table S6: Gene Ontology analysis of the DEGs. [file 2923441.f6.pdf]

Supplementary Table S6. Gene Ontology analysis of the DEGs.

ID	Term	Count	*P*-value	Genes
**Biological Process**				
GO:0006955	immune response	22	3.02E-09	IL1R2, GZMA, SLC11A1, AQP9, NCF4, C5AR1, HLA-C, LTB4R, GZMH, ZAP70, FCGR3B, IL18RAP, NFIL3, CD8A, BPI, PGLYRP1, TLR4, LAT, IL18R1, HLA-DQA1, HLA-DRB1, TLR2
GO:0045087	innate immune response	20	1.22E-07	CR1, NCF1, HMGB2, MATK, HLA-C, BMX, TREM1, ZAP70, GZMM, CLEC4D, S100A12, PADI4, PGLYRP1, CLEC4E, CD46, TLR4, CD55, CAMP, KLRG1, TLR2
GO:0006954	inflammatory response	18	4.75E-07	ORM1, SLC11A1, C5AR1, FPR1, FOS, PTGS2, LTB4R, NDST1, TPST1, ZAP70, MMP25, IL18RAP, PROK2, S100A12, TLR4, LAT, KLRG1, TLR2
GO:0032496	response to lipopolysaccharide	10	5.60E-05	SLC11A1, C5AR1, PELI1, HMGB2, ALPL, IRAK3, FOS, PTGS2,

				TLR4, TLR2
GO:0042742	defense response to bacterium	8	8.16E-04	CLEC4D, GNLY, SLC11A1, BCL3, S100A12, CLEC4E, TLR4, CAMP
GO:0006935	chemotaxis	7	0.001753706	CX3CR1, C5AR1, PLAUR, FPR1, PROK2, CMTM2, CXCL16
GO:0002250	adaptive immune response	7	0.004576779	EOMES, ZAP70, CLEC4D, CD46, LILRA2, LILRA3, LAT
GO:0045730	respiratory burst	6	1.40E-07	CD52, NCF1, SLC11A1, NCF1B, NCF1C, CD55
GO:0050830	defense response to Gram-positive bacterium	6	0.001944214	C5AR1, HMGB2, PGLYRP1, CAMP, HIST2H2BE, TLR2
GO:0071222	cellular response to lipopolysaccharide	6	0.006593787	CX3CR1, ARG1, HMGB2, TLR4, CAMP, CXCL16
GO:0007204	positive regulation of cytosolic calcium ion concentration	6	0.013188023	CD52, C5AR1, FPR1, PROK2, UTS2, CD55
GO:0050776	regulation of immune response	6	0.038629645	KLRB1, CD8A, HLA-C, ITGB7, TREM1, CD3D
GO:0030890	positive regulation of B cell proliferation	5	7.05E-04	BST1, PELI1, IRS2, TLR4, ADA
GO:0050829	defense response to Gram-negative bacterium	5	0.002569748	SLC11A1, HMGB2, BPI, TLR4, CAMP
GO:0019882	antigen processing and presentation	5	0.002569748	CD8A, RAB27A, HLA-C, HLA-DRB1, HLA-DQA1

GO:0006968	cellular defense response	5	0.003973065	CX3CR1, NCF1, GNLY, C5AR1, KLRG1
GO:0046718	viral entry into host cell	5	0.009751936	CR1, ANPEP, ITGB7, CD46, CD55
GO:0043085	positive regulation of catalytic activity	5	0.010176964	NCF1, ALOX5AP, NCF4, NCF1B, NCF1C
GO:0007169	transmembrane receptor protein tyrosine kinase signaling pathway	5	0.01803326	ZAP70, CD8A, MATK, GAB2, BMX
GO:0045766	positive regulation of angiogenesis	5	0.032292487	CX3CR1, LRG1, C5AR1, UTS2, CAMP
GO:0050900	leukocyte migration	5	0.038828473	C5AR1, FPR1, CD58, TREM1, MMP9
GO:0042346	positive regulation of NF-kappaB import into nucleus	4	0.00128885	PTGS2, TLR4, IL18R1, TLR2
GO:0032715	negative regulation of interleukin-6 production	4	0.003009417	ORM1, IRAK3, BPI, TLR4
GO:0030449	regulation of complement activation	4	0.003673224	CR1, C5AR1, CD46, CD55
GO:0032720	negative regulation of tumor necrosis factor production	4	0.007182176	ORM1, IRAK3, BPI, TLR4
GO:0032729	positive regulation of interferon-gamma	4	0.012164594	SLC11A1, BCL3, TLR4, IL18R1

	production			
GO:0042110	T cell activation	4	0.01289626	ZAP70, CD8A, LAT, ADA
GO:0060326	cell chemotaxis	4	0.030346256	NOV, C5AR1, FPR1, HMGB2
GO:0045454	cell redox homeostasis	4	0.046473672	NCF1, SLC11A1, NCF4, CAMP
GO:0042088	T-helper 1 type immune response	3	0.006654696	BCL3, TLR4, HLA-DRB1
GO:0006691	leukotriene metabolic process	3	0.010372297	ALOX5AP, CYP4F3, TLR2
GO:0045664	regulation of neuron differentiation	3	0.018140886	EOMES, BIN1, CDK5R1
GO:0050850	positive regulation of calcium-mediated signaling	3	0.018140886	ZAP70, CD8A, ADA
GO:0006801	superoxide metabolic process	3	0.018140886	NCF1, NCF1B, NCF1C
GO:0019835	cytolysis	3	0.019914784	GZMM, GZMA, GZMH
GO:0045987	positive regulation of smooth muscle contraction	3	0.019914784	PROK2, PTGS2, ADA
GO:0032733	positive regulation of interleukin-10 production	3	0.023669402	CD46, TLR4, TLR2
GO:0045742	positive regulation of epidermal growth factor receptor signaling pathway	3	0.025646901	HIP1, PLAUR, MMP9
GO:0010575	positive regulation of vascular endothelial growth	3	0.031961368	C5AR1, PTGS2, SULF2

	factor production			
GO:0032728	positive regulation of interferon-beta production	3	0.031961368	HMGB2, TLR4, TLR2
GO:0043388	positive regulation of DNA binding	3	0.034188441	PLAUR, HMGB2, MMP9
GO:0032689	negative regulation of interferon-gamma production	3	0.034188441	PGLYRP1, TLR4, HLA-DRB1
GO:0050873	brown fat cell differentiation	3	0.043668861	RGS2, LRG1, PTGS2
GO:0002755	MyD88-dependent toll-like receptor signaling pathway	3	0.046174928	IRAK3, TLR4, TLR2
GO:0002374	cytokine secretion involved in immune response	2	0.030942012	TREM1, TLR2
GO:0002292	T cell differentiation involved in immune response	2	0.030942012	CLEC4D, CLEC4E
GO:0032613	interleukin-10 production	2	0.030942012	CD46, TLR2
GO:0045916	negative regulation of complement activation	2	0.030942012	CD46, CD55
GO:0038094	Fc-gamma receptor signaling pathway	2	0.04104298	CLEC4D, CLEC4E
**Cellular Component**				
GO:0016021	integral component of membrane	77	1.24E-04	ST6GALNAC2, AQP9, DYSF, SLC40A1, GIMAP5, CD3D, TREM1, TMEM91,

				LTB4R, CXCL16, NDST1, PANX2, MMP25, FCGR3B, SLC22A15, IL18RAP, ANPEP, CA4, ITGB7, DIRC2, SLC37A3, LBR, CD52, CR1, APAF1, MME, ACSL1, SLC11A1, IL1R2, PLAUR, FLT3LG, HLA-C, MCEMP1, CKAP4, TPST1, CLEC4D, CD8A, CEACAM4, BPI, CLEC4E, CD46, RGR, TLR2, B4GALT5, SLC22A4, CX3CR1, RTN3, KLRB1, FPR1, LILRA2, TMEM71, SDCBP, ZDHHC18, CHST13, CD58, KIAA0319L, TP53I11, HLA-DQA1, KLRG1, MGAM, CYP4F3, KCNJ15, NKG7, TM6SF1, RNF149, CPD, ALOX5AP, SLC26A8, ALPL, MANSC1, CMTM2, ITM2A, LAT, HLA-DRB1, IL18R1, MEGF9, SIGLEC5
GO:0005886	plasma membrane	71	1.95E-06	AQP9, DYSF, SLC40A1, IRS2, PYGL, CD3D, TREM1, LTB4R, CXCL16,

				PANX2, MMP25, RGS2, FCGR3B, IL18RAP, CA4, ITGB7, CR1, MME, ACSL1, SLC11A1, IL1R2, PLAUR, HLA-C, GAB2, OLFM4, FAM126B, CKAP4, F5, ZAP70, CLEC4D, CD8A, ACOX1, RP2, CLEC4E, CD46, TLR4, TLR2, RGS18, SLC22A4, CX3CR1, RTN3, KLRB1, C5AR1, FPR1, LILRA3, SDCBP, GNG10, S100A12, CD58, KIAA0319L, HLA-DQA1, CD55, KLRG1, MGAM, GCA, KCNJ15, SULF2, BST1, VNN2, VNN3, STK17B, CPD, IL2RB, SLC26A8, ALPL, ITM2A, LAT, HLA-DRB1, ADA, IL18R1, CDK5R1
GO:0070062	extracellular exosome	50	6.99E-05	HIST2H2AA3, ORM1, DYSF, PYGL, FCGR3B, ANPEP, CA4, ITGB7, PGLYRP1, TGM3, CAMP, CR1, APAF1, MME, ARG1, PLAUR, HLA-C,

				OLFM4, MMP9, HIST2H2BE, CKAP4, CRISPLD2, RP2, BOLA2, BPI, CD46, FKBP5, CREB5, B4GALT5, RTN3, CSTA, NCALD, SDCBP, GNG10, CD58, KIAA0319L, CD55, MGAM, NQO2, GCA, RAB27A, BST1, LRG1, RNF149, QPCT, CPD, HIST1H4H, ALPL, ITM2A, HLA-DRB1
GO:0016020	membrane	41	1.62E-04	HIP1, NCF4, CXCL16, LMNB1, SDCBP, MMP25, CA4, ITGB7, CD58, LBR, HLA-DQA1, CD52, HSDL2, ACSL1, SLC11A1, KCNJ15, FLT3LG, HLA-C, FOS, CKAP4, F5, GZMH, TPST1, GZMM, LRG1, RNF149, BIN1, ACOX1, CPD, CEACAM4, IL2RB, ALOX5AP, SLC26A8, HIST1H4H, ALPL, FOLR3, LAT, ADA, HLA-DRB1, CDK5R1, FKBP5
GO:0005887	integral component	29	4.98E-04	CX3CR1, SLC22A4, C5AR1,

	of plasma membrane			AQP9, SLC40A1, FPR1, LTB4R, ANPEP, CD58, CD55, HLA-DQA1, CD52, CR1, MME, SLC11A1, PLAUR, KCNJ15, NKG7, HLA-C, CD8A, CEACAM4, IL2RB, SLC26A8, BPI, CD46, RGR, TLR4, HLA-DRB1, TLR2
GO:0005576	extracellular region	28	0.006559638	CDA, ORM1, PSG11, LILRA2, TREM1, LILRA3, CXCL16, PROK2, S100A12, PGLYRP1, CD55, CAMP, IL1R2, GZMA, FLT3LG, HLA-C, MMP9, F5, GZMK, GZMM, LRG1, NOV, CRISPLD2, CD8A, TCN1, UTS2, HIST1H4H, FOLR3
GO:0005615	extracellular space	26	0.002455719	RTN3, CSTA, ORM1, HMGB2, CXCL16, SDCBP, GNLY, ANPEP, CAMP, ARG1, FLT3LG, OLFM4, PRSS33, MMP9, HIST2H2BE, SULF2, F5, LRG1, TCN1, VNN3, CPD, UTS2,

GO:0009986	cell surface	13	0.009500746	ALPL, BPI, CMTM2, ADA CR1, C5AR1, HLA-C, SULF2, CA4, ITGB7, CD58, CD46, TLR4, ADA, CD55, HLA-DRB1, TLR2
GO:0000139	Golgi membrane	12	0.039091233	RTN3, NDST1, TPST1, ST6GALNAC2, HLA-C, CHST13, KIAA0319L, HLA-DRB1, CD55, HLA-DQA1, B4GALT5, F5
GO:0031225	anchored component of membrane	11	2.29E-07	CD52, BST1, MMP25, FCGR3B, VNN2, VNN3, CA4, PLAUR, ALPL, CD58, CD55
GO:0043020	NADPH oxidase complex	4	2.12E-04	NCF1, NCF4, NCF1B, NCF1C
GO:0031234	extrinsic component of cytoplasmic side of plasma membrane	4	0.031882704	ZAP70, MATK, BMX, TGM3
GO:0042101	T cell receptor complex	3	0.014093588	ZAP70, CD8A, CD3D
GO:0042613	MHC class II protein complex	3	0.020722333	HLA-C, HLA-DRB1, HLA-DQA1
GO:0071556	integral component of lumenal side of	3	0.034778815	HLA-C, HLA-DRB1, HLA-DQA1

	endoplasmic reticulum membrane			
GO:0001772	immunological synapse	3	0.046506321	ZAP70, GZMA, LAT
GO:0032010	phagolysosome	2	0.039994899	NCF1, NCF4
**Molecular Function**				
GO:0004872	receptor activity	14	3.30E-07	SLC40A1, PLAUR, LILRA2, TR
GO:0042803	protein homodimerization activity	14	0.034737976	CDA, HIP1, GCA, CEBPD, GZM
GO:0046982	protein heterodimerization activity	12	0.007876517	HIST2H2AA3, SDCBP, HIP1, G
GO:0003824	catalytic activity	9	6.69E-04	HAL, MGAM, PFKFB3, ACSM3,
GO:0030246	carbohydrate binding	7	0.014905314	MGAM, CLEC4D, KLRB1, CLC,
GO:0004252	serine-type endopeptidase activity	7	0.045669734	GZMK, GZMM, GZMA, PRSS33
GO:0001618	virus receptor activity	6	7.16E-04	CR1, ANPEP, ITGB7, CD46, HL
GO:0043325	phosphatidylinositol-3,4-bisphosphate binding	5	1.09E-04	HIP1, NCF1, NCF1B, NCF1C, G
GO:0035091	phosphatidylinositol binding	5	0.010898769	HIP1, NCF1, NCF4, NCF1B, NC
GO:0016176	superoxide-generating NADPH oxidase activator activity	4	1.16E-04	NCF1, NCF4, NCF1B, NCF1C
GO:0016175	superoxide-generating NADPH oxidase activity	3	0.005283765	NCF1, NCF1B, NCF1C
GO:0050786	RAGE receptor binding	3	0.005283765	FPR1, HMGB2, S100A12
GO:0032395	MHC class II receptor activity	3	0.009822768	HLA-C, HLA-DRB1, HLA-DQA
GO:0001530	lipopolysaccharide binding	3	0.020632864	BPI, TLR4, TLR2
GO:0045296	cadherin binding	3	0.022449496	OLFM4, CD46, CDK5R1

GO:0042605	peptide antigen binding	3	0.032456863	HLA-C, HLA-DRB1, HLA-DQA1
GO:0032266	phosphatidylinositol-3-phosphate binding	3	0.041488027	HIP1, PLEKHF1, NCF4
GO:0017159	pantetheine hydrolase activity	2	0.030084164	VNN2, VNN3
GO:0001875	lipopolysaccharide receptor activity	2	0.049638648	TLR4, TLR2
