# Supplementary material for: MicroRNA-146a Serves as a Biomarker for Adverse Prognosis of ST-Segment Elevation Myocardial Infarction
Source: Cardiovasc Ther. 2021 Oct 25;2021:2923441. doi: 10.1155/2021/2923441 (PMC8561321; doi:10.1155/2021/2923441)
Supplement: Supplementary 7 — Supplementary Table S7: Kyoto Encyclopedia of Genes and Genomes pathway analysis of the DEGs. [file 2923441.f7.docx]

| Supplementary Table S7. Kyoto Encyclopedia of Genes and Genomes pathway analysis of the DEGs | | | | |
| --- | --- | --- | --- | --- |
| ID | Term | Count | *P*-value | Genes |
| hsa05140 | Leishmaniasis | 10 | 2.36E-07 | CR1, FCGR3B, NCF1, |
|  |  |  |  | NCF4, FOS, PTGS2, |
|  |  |  |  | TLR4, HLA-DRB1, |
|  |  |  |  | HLA-DQA1, TLR2 |
| hsa04640 | Hematopoietic cell lineage | 9 | 1.33E-05 | CR1, MME, CD8A, |
|  |  |  |  | ANPEP, IL1R2, FLT3LG, |
|  |  |  |  | CD3D, HLA-DRB1, CD55 |
| hsa05321 | Inflammatory bowel | 6 | 0.001287211 | IL18RAP, TLR4, HLA-DRB1, |
|  | disease (IBD) |  |  | IL18R1, HLA-DQA1, TLR2 |
| hsa04610 | Complement and | 6 | 0.001804852 | CR1, C5AR1, PLAUR, |
|  | coagulation cascades |  |  | CD46, CD55, F5 |
| hsa05152 | Tuberculosis | 9 | 0.001824963 | CR1, FCGR3B, APAF1, |
|  |  |  |  | CLEC4E, TLR4, HLA-DRB1, |
|  |  |  |  | CAMP, HLA-DQA1, TLR2 |
| hsa04145 | Phagosome | 8 | 0.002957786 | FCGR3B, NCF1, NCF4, |
|  |  |  |  | HLA-C, TLR4, HLA-DRB1, |
|  |  |  |  | HLA-DQA1, TLR2 |
| hsa05150 | Staphylococcus aureus | 5 | 0.004859057 | FCGR3B, C5AR1, FPR1, |
|  | infection |  |  | HLA-DRB1, HLA-DQA1 |
| hsa04380 | Osteoclast differentiation | 7 | 0.006438557 | FCGR3B, NCF1, NCF4, |
|  |  |  |  | FOS, GAB2, LILRA2, LILRA3 |
| hsa05340 | Primary immunodeficiency | 4 | 0.009092664 | ZAP70, CD8A, CD3D, ADA |
| hsa04668 | TNF signaling pathway | 6 | 0.011699259 | BCL3, FOS, PTGS2, MMP9, |
|  |  |  |  | IL18R1, CREB5 |
| hsa05144 | Malaria | 4 | 0.02441473 | CR1, KLRB1, TLR4, TLR2 |
| hsa05323 | Rheumatoid arthritis | 5 | 0.025844695 | FOS, TLR4, HLA-DRB1, |
|  |  |  |  | HLA-DQA1, TLR2 |
| hsa05322 | Systemic lupus | 6 | 0.028150382 | HIST2H2AA3, FCGR3B, |
|  | erythematosus |  |  | HIST1H4H, HLA-DRB1, |
|  |  |  |  | HLA-DQA1, HIST2H2BE |
| hsa05134 | Legionellosis | 4 | 0.031412058 | CR1, APAF1, TLR4, TLR2 |
| hsa04514 | Cell adhesion | 6 | 0.034927149 | CD8A, HLA-C, ITGB7, |
|  | molecules (CAMs) |  |  | CD58, HLA-DRB1, |
|  |  |  |  | HLA-DQA1 |
| hsa05416 | Viral myocarditis | 4 | 0.036062991 | HLA-C, HLA-DRB1, |
|  |  |  |  | CD55, HLA-DQA1 |
| hsa05161 | Hepatitis B | 6 | 0.037708456 | APAF1, FOS, TLR4, |
|  |  |  |  | MMP9, CREB5, TLR2 |
| hsa04660 | T cell receptor | 5 | 0.038767373 | ZAP70, CD8A, FOS, |
|  | signaling pathway |  |  | CD3D, LAT |
